# Clinical Application of Pharmacogenetic Markers in the Treatment of Dermatologic Pathologies

**DOI:** 10.3390/ph14090905

**Published:** 2021-09-06

**Authors:** Cristina Membrive Jiménez, Cristina Pérez Ramírez, Almudena Sánchez Martín, Sayleth Vieira Maroun, Salvador Arias Santiago, María Carmen Ramírez Tortosa, Alberto Jiménez Morales

**Affiliations:** 1 Pharmacy Service, Pharmacogenetics Unit, University Hospital Virgen de las Nieves, 18014 Granada, Spain; cristina.membrive95@gmail.com (C.M.J.); almuweb06@gmail.com (A.S.M.); saylethvieira@gmail.com (S.V.M.); alberto.jimenez.morales.sspa@juntadeandalucia.es (A.J.M.); 2 Center of Biomedical Research, Department of Biochemistry and Molecular Biology II, Institute of Nutrition and Food Technology “José Mataix”, University of Granada, Avda. del Conocimiento s/n., Armilla, 18016 Granada, Spain; mramirez@ugr.es; 3 Dermatology Service, University Hospital Virgen de las Nieves, 18016 Granada, Spain; salvadorarias@ugr.es

**Keywords:** dermatology, polymorphisms, mutations, response, toxicity, biologic therapy

## Abstract

Dermatologic pathologies are the fourth most common cause of non-fatal disease worldwide; however, they produce a psychosocial, economic, and occupational impact equal to or greater than other chronic conditions. The most prevalent are actinic keratosis, followed by basal-cell carcinoma, in a lesser proportion acne vulgaris, psoriasis, and hidradenitis suppurativa, among others, and more rarely dermatitis herpetiformis. To treat actinic keratosis and basal-cell carcinoma, 5-fluorouracil (5-FU) 0.5% is administered topically with good results, although in certain patients it produces severe toxicity. On the other hand, dapsone is a drug commonly used in inflammatory skin conditions such as dermatitis herpetiformis; however, it occasionally causes hemolytic anemia. Additionally, biologic drugs indicated for the treatment of moderate-to-severe psoriasis and hidradenitis suppurativa have proved to be effective and safe; nevertheless, a small percentage of patients do not respond to treatment with biologics in the long term or they are ineffective. This interindividual variability in response may be due to alterations in genes that encode proteins involved in the pathologic environment of the disease or the mechanism of action of the medication. Pharmacogenetics studies the relationship between genetic variations and drug response, which is useful for the early identification of non-responsive patients and those with a higher risk of developing toxicity upon treatment. This review describes the pharmacogenetic recommendations with the strongest evidence at present for the treatments used in dermatology, highlighting those included in clinical practice guides. Currently, we could only find pharmacogenetic clinical guidelines for 5-FU. However, the summary of product characteristics for dapsone contains a pharmacogenetic recommendation from the United States Food and Drug Administration. Finally, there is an enormous amount of information from pharmacogenetic studies in patients with dermatologic pathologies (mainly psoriasis) treated with biologic therapies, but they need to be validated in order to be included in clinical practice guides.

## 1. Introduction

Skin diseases are among the most prevalent reasons for seeking health care, varying between 5.5% and 22.5%. Most are not life-threatening; however, the psychosocial, economic, and occupational impact of these diseases is considered to be equal to or greater than other chronic pathologies [1]. In particular, 25% of dermatologic pathologies can even cause disability [2]. They are classified as primary skin diseases, cutaneous manifestations of systemic diseases, or sexually transmitted diseases (such as genital herpes) [3]. In Europe, the most common are warts, acne, contact dermatitis, psoriasis, and vitiligo [4].

Dermatologic treatments vary according to the pathology and its severity. Topically administered drugs (such as topical corticosteroids) and physical therapy (heat, cold, laser radiation, electromagnetic radiation) are mainly used. However, the most severe conditions require systemic drugs, biologic therapies, and/or surgical treatments [5]. Systemic and biologic drug therapies are generally effective and safe, but certain patients do not respond in the short or long-term and/or show various degrees of toxicity [6].

The need to find predictive and prognostic biomarkers to guide the choice of dermatologic treatment leads to the implementation of pharmacogenetics (PGx). This is a tool that enables the early identification of patients with a greater therapeutic benefit and lower risk of undergoing adverse events or suffering toxicity from treatment [7]. Currently, PGx research in the area of dermatology is steadily growing. In recent years, an enormous amount of genetic data has been generated from large cohorts of patients with a range of clinical phenotypes [8]. Consequently, these pharmacological treatment patterns based on genetic diversity have been transferred to PGx clinical guidelines or recommendations by the European Medicines Agency (EMA) and the United States Food and Drug Administration (FDA) [9].

On the other hand, the importance of PGx is also particularly marked in dermatology owing to the variety of systemic drugs indicated in other pathologies that may develop toxicity with severe cutaneous manifestations [10]. As a result, PGx recommendations in dermatology are crucial to avoid the development of cutaneous adverse reactions and to maximize therapeutic impact [11]. The implementation of PGx in regular clinical practice is a very important challenge at present for the health care system. The interpretation and understanding of PGx studies, the recommendations of the health care authorities, and PGx clinical guidelines will be the basis for applying PGx in regular dermatologic clinical practice, thereby improving the patient’s quality of life by establishing personalized medicine [12].

**Figure 1:**
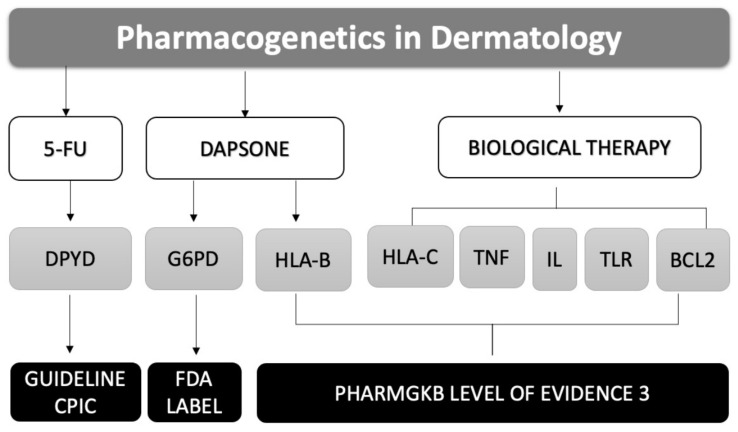
Graphical abstract of pharmacogenetics in clinical dermatology.

This literature review presents the information currently available on pharmacogenetically significant drugs used to treat skin diseases. In particular, it indicates the recommendations in PGx clinical guidelines for 5-fluorouracil, specifically when used topically in patients diagnosed with actinic keratosis and/or basal-cell carcinoma, and the PGx recommendation in the summary of product characteristics for the drug dapsone when administered systemically to treat dermatitis herpetiformis. Finally, it highlights the most important PGx studies on biologic therapies indicated for hidradenitis suppurativa and moderate-to-severe psoriasis (Figure 1).

## 2. Materials and Methods

A PubMed search included key words “dermatology”, “clinical”, “CPIC” together with “treatment”, and “polymorphisms” and “response” or “toxicity”. Data regarding gene, SNP, drug, pathology, population, level of evidence PharmGKB, clinical application, allele, phenotype category (response or toxicity), year of publication, number of patients were recorded.

## 3. Pharmacogenetically Significant Dermatologic Pathologies

### 3.1. Actinic Keratosis

Actinic keratoses (AKs) are in situ SCC due to an abnormal intraepidermal proliferation of atypical keratinocytes, through a chronic exposure to sunlight, and may develop invasive squamous cell carcinoma, although this definition is still being studied [13]. They manifest as erythematous macules or papules with rough surfaces and, in some cases, with hyperkeratosis, which usually appear on the face, the alopecic scalp, and the back of the hands, on skin surfaces continually exposed to solar radiation [14]. Actinic keratosis is one of the main reasons for dermatologic consultations and, because of its increasing global prevalence, it is estimated to be the most common in situ skin carcinoma (15). In Europe, the estimated prevalence in the population over the age of 70 is 34% in men and 18% in women [13]. Even so, some 30–42% of patients with AK ignore these lesions and, consequently, it continues to be underdiagnosed. It commonly develops in light-skinned men over the age of 45 with a high degree of exposure to sunlight and/or artificial ultraviolet radiation [15].

The therapeutic approach to AKs is based on two objectives: treating the lesions in isolation and field cancerization. The choice of one or the other will depend on patient characteristics, location, number of lesions, previous treatments, adherence, practitioner experience, and drug efficacy [16]. The treatment of isolated lesions consists of cryotherapy, electrocoagulation, imiquimod, and topical 5-fluorouracil 0.5% with salicylic acid 10%. However, if the lesions become malignant, the treatment is based on 5-fluorouracil (5-FU), photodynamic therapy, imiquimod, and diclofenac 3% in hyaluronic acid gel 2.5%. In people with chronic actinic damage, a combination of the treatments is recommended, together with strict photoprotection [14].

### 3.2. Basal-Cell Carcinoma

Basal-cell carcinoma (BCC) is the most common malignant cutaneous tumor in Caucasians, accounting for approximately 80–90% of skin cancers [17,18]. It forms from cells similar to those of the basal level of the epidermis and the cutaneous appendages, it is slow-growing, and it does not usually produce metastases [19]. The possible causes of this pathology mainly include exposure to sunlight and radiation, age, and skin type [20]. The lesions are usually located on the face, especially in the area of the medial canthus of the eye, the nose, and the forehead. The initial lesion is normally a shiny, pearly papule surrounded by small blood vessels. It can reach a considerable size and although it does not usually metastasize, if left untreated it may give rise to large-scale tissue destruction [19,21].

The treatment objective is the complete elimination of the tumor and maximum functional and cosmetic preservation of the affected area. The choice of treatment depends on the clinical and histopathologic features influencing the risk of lesion recurrence [21]. Basal-cell carcinomas with a high risk of recurrence are treated primarily with surgery; less frequently, however, topical 5-FU or imiquimod, cryotherapy, intralesional injection, and photodynamic therapy are also used [21].

### 3.3. Dermatitis Herpetiformis

Dermatitis herpetiformis (DH) is a chronic, polymorphous, pruriginous condition associated with celiac disease. The characteristic cutaneous manifestations are papules with vesicles grouped around an erythematous base with excoriations, crusts, and occasional lichenification due to scratching, symmetrically distributed over the extensor surfaces of the elbows and knees, as well as the back, scalp and buttocks [22,23]. It is mainly found in Caucasian men at any age and is rare in children. The incidence varies by geographic location and a familial incidence of around 2.3–6.5% has been observed [24]. In various studies conducted in Europe and North America, the DH incidence figures vary between 0.4 and 3.5 per 100,000 per year [25].

The first-line treatment for DH and the only therapy that can improve the intestinal disease is a strict gluten-free diet. However, dapsone is the main medical treatment capable of inducing an acute response in DH eruptions in 24 h. Sulfonamides can also be used to control the acute phase of the disease; the most commonly prescribed drugs are sulfapyridine and sulfamethoxypyridazine [22,26].

### 3.4. Psoriasis

Psoriasis is a chronic and recurrent inflammatory autoimmune disease with a worldwide prevalence of up to 8.5% in adults and 2.1% in children [27]. The skin manifestations are not life-threatening, apart from exceptional cases of erythrodermic or pustular psoriasis. However, it has a severe impact on patients’ quality of life and generates high health-care costs. Furthermore, it is associated with other pathologies and is, therefore, regarded as a systemic entity rather than an exclusively dermatologic disease [28]. Its etiology is unclear, although it is thought that it could be due to a combination of genetic, immunologic, and environmental factors (such as stress, trauma, medications, and microbial infections, among others) [29]. It has been found that the incidence differs between ethnicities and is greater among relatives and even more between monozygotic twins [30]. The presence of the HLA-C*06:02 allele has been described as a factor in susceptibility to psoriasis due to its regulatory function in autoimmunity against melanocytes [31].

Psoriatic lesions are produced and maintained by alterations in cutaneous immune responses, mediated by dendritic cells activated by Toll-like receptors, producing a cascade of cytokines (TNFα, IL-17, IL-23 and IL-12), which trigger the hyperproliferation of keratinocytes in the epidermis and give rise to the appearance of epidermal hyperplasia typical of psoriasis [32]. Ninety percent of patients develop clinical manifestations in the form of erythematous plaques covered by whitish scales on the scalp, elbows, knees, and back. The severity of the lesions is measured by the psoriasis area severity index (PASI), body surface area (BSA), and dermatology life quality index (DLQI) indicators. Moderate-to-severe psoriasis is considered to be psoriasis with PASI > 10, BSA > 10, and DLQI > 10. The effectiveness of treatment is evaluated by absolute PASI values or percentage improvement in PASI; for example, a 90% reduction in the lesions (PASI90) [33].

The treatments used are aimed at blocking the inflammatory response and depend on the severity of the psoriasis; topicals, phototherapy, classic systemic immunomodulators, or biologic therapy may be used. In cases of moderate-to-severe psoriasis, systemic therapy (methotrexate, cyclosporine, acitretin, apremilast, fumaric acid esters), phototherapy, or photochemotherapy are recommended, and as a last option, when there is no response to previous treatments or they are contraindicated, biologic therapy is used [34].

There is a wide range of biologic treatments indicated for moderate-to-severe psoriasis. They are classified into two groups: tumor necrosis factor inhibitor (anti-TNF) therapies, such as infliximab (INF), etanercept (ETN), adalimumab (ADA), and certolizumab (CTL), and cytokine inhibitors such as ustekinumab (UTK), secukinumab (SCK), ixekizumab (IXE), brodalumab (BDL), guselkumab (GSL), tildrakizumab (TDK), and risankizumab (RSK) [35].

### 3.5. Hidradenitis Suppurativa

Hidradenitis suppurativa (HS), also known as acne inversa, is a chronic, inflammatory, recurrent, debilitating skin disease affecting the hair follicles. It is relatively common, with a prevalence of 0.05% to 4.1% [36]. It usually appears after puberty with painful, deep-seated, inflamed lesions in areas of the body containing apocrine glands, most commonly in the axillary, inguinal, and anogenital regions [37]. Moreover, it is associated with various concomitant or secondary conditions, including obesity, metabolic syndrome, Crohn’s disease, rheumatic pathologies, and squamous cell carcinoma [38]. Its physical and psychological impact makes HS the dermatologic condition that most impairs the patient’s quality of life [36,39]. Its etiology is unknown, although it is suspected that there is a genetic component with a probable hormonal influence [40]. Although triggering factors such as obesity and smoking strongly affect the development of HS, genetic influence is crucial [38]. In particular, it has been observed that 5% of patients with HS have a mutation in the gamma-secretase enzyme in the Notch signaling pathway involved in the epidermal differentiation and proliferation processes, as well as in maintaining the integrity of the hair follicle and sebaceous glands. These mutations are acquired by autosomal dominant inheritance and are associated with the severe and extensive phenotype. Furthermore, 42% of patients who develop HS have a family history [41].

Severe forms of the disease are marked by tissue destruction and scarring phenomena with the formation of subcutaneous tunnels or fistulas. An evaluation of the severity of HA is based on lesion morphology (nodules, abscesses, tunnels, and scars), location (axillae, inframammary folds, groin, perigenital, or perineal), and lesion progression (two recurrences within 6 months or chronic or persistent lesions for 3 months or more) [42,43]. The Hurley staging system enables us to classify patients into three stages: one (solitary or multiple isolated abscesses with no scarring or fistula tracts), two (recurrent abscesses, single or multiple widely separated lesions, with fistula tract formation), and three (diffuse or extensive involvement, with multiple interconnected fistula tracts and abscesses). The clinical treatment response can be measured with various indicators; the most widely used in pharmacological studies to evaluate the response is HiSCR (Hidradenitis Suppurativa Clinical Response). This is defined as a reduction of 50% or more in the inflammatory lesion count (inflammatory nodules plus abscesses) with no increase in the number of abscesses or the number of inflammatory fistulas [44].

The management of this pathology comprises various guidelines: general measures to reduce the bacterial load (antiseptic soaps, warm baths, and so on), pharmacological therapy (topical, intralesional, and systemic), surgery (including direct closure, second-intention healing, grafts, and flaps), and other measures (CO_2_ laser treatment, radiotherapy, etc.). Pharmacological treatment depends on the severity and proves difficult in many cases due to a typically poor response to the various treatments. However, it is important to treat this pathology because it may present serious complications in the long term [40]. The European guideline recommends matching the treatment of HS to the Hurley severity stage according to the following treatment algorithm: cases of mild HS are treated with local excision, laser treatment, and topical clindamycin; however, in patients with more extensive and severe HS, radical surgical excision with systemic antibiotic or retinoid treatment is recommended. In some patients, a concomitant or sequential use of various drugs is sufficient to control the inflammatory load in the medium and long-term; other cases, however, require making use of biologic therapy [45]. Specifically, ADA is the only biologic so far approved by the FDA and the EMA for the treatment of moderate-to-severe HS [44,46]. Furthermore, because fistulas tend to be refractory to medical treatment, once structural damage has already occurred, various surgical techniques need to be used to resolve it [42,47].

## 4. General Pharmacogenetics of the Disease

This section describes the current PGx findings in the treatment of dermatologic pathologies. It is classified into three levels according to the type of treatment: topical treatment: 5-FU in actinic keratosis and basal-cell carcinoma; systemic treatment: dapsone in dermatitis herpetiformis; finally, treatment with biologic therapies in moderate-to-severe psoriasis and hidradenitis suppurativa (Table 1).

### 4.1. Topical Treatment: 5-FU in Actinic Keratosis and Basal-Cell Carcinoma

5-Fluorouracil (5-FU) is a parenterally administered pyrimidine analogue, indicated for the antineoplastic treatment of various types of cancer, but also used in dermatology to treat AK and BCC, administered topically at a low concentration (0.5%) and with salicylic acid (to contribute to its keratolytic effect and enhance the penetration of the drug through the epidermis) [48]. This medication is an analogue of pyrimidine, which interferes in the synthesis of DNA and RNA, blocking the conversion of deoxyuridilic acid to thymidylic acid by the irreversible inhibition of the enzyme thymidylate synthetase. It also produces alterations in the complex that regulates RNA degradation and preferentially affects cells with a high replication rate, causing toxicity and cell death [49].

It is metabolized by the enzyme dihydropyrimidine dehydrogenase (DPD), generating an inactive metabolite called dihydrofluorouracil; this regulates plasma concentrations of 5-FU [50]. Of the dose administered, 85% is rapidly eliminated, so the action of DPD is particularly important for determining the patient’s treatment response [51,52]. The DPD enzyme is encoded by the *DPYD* gene located on chromosome 1p21.3 and alterations in this gene lead to a reduction in enzyme activity; consequently, producing an increase in the half-life of 5-FU and, therefore, a greater risk of toxicity [53]. Around 3–5% of individuals have alterations of the *DPYD* gene and a deficiency in this enzyme and, therefore, present a higher risk of toxicity through the use of 5-FU [51,52].

Numerous genetic variants of *DPYD* have been identified, the most important being rs3918290 (G > A), rs55886062 (T > G), rs67376798 (A > T), and rs75017182 (C > G), due to the fact that they generate a significant reduction in enzymatic activity and increase the risk of toxicity [50]. Of these variants, rs3918290 and rs55886062 have the most harmful impact on DPD, while rs67376798 and rs75017182 result in moderately reduced DPD activity [50].

### 4.2. Systemic Treatment: Dapsone in Dermatitis Herpetiformis

Dapsone is a sulfonamide widely used in dermatology for its anti-inflammatory properties. In particular, patients with DH use it orally at doses of 50–100 mg/24 h to rapidly reduce the acute reaction [54]. Recently, moreover, dapsone administered topically at low concentrations has received exceptional approval for the treatment of acne vulgaris in the United States and Canada [55,56].

Its anti-inflammatory effect is similar to that of non-steroidal anti-inflammatory drugs (NSAIDs). Furthermore, it shows antimicrobial and antiprotozoal properties and is, therefore, also indicated for treating infectious diseases, such as malaria [54,57]. Dapsone administered orally is absorbed in the gastrointestinal tract with high bioavailability. After absorption, it is metabolized both in the liver and by activated polymorphonuclear leukocytes (PMNs) or mononuclear cells. In the liver, dapsone is acetylated by the N-acetyltransferase enzyme and through cytochrome P-450 hydroxylation a metabolite called dapsone hydroxylamine is produced. The acetylation speed is genetically determined and there are patients who are slow or fast metabolizers. In slow metabolizers, toxic effects are observed, mainly due to dapsone hydroxylamine [58]. This metabolite can cause the oxidation of red blood cells, leading to its main hematologic side effect, hemolytic anemia. These effects are usually greater in the absence of a protective mechanism generated by reduced nicotinamide adenine dinucleotide phosphate (NADPH), mostly produced by the erythrocyte enzyme glucose-6-phosphate dehydrogenase (G6PD). On this basis, individuals with G6PD deficiency are approximately twice as sensitive to dapsone-induced hemolytic anemia [59].

In addition, dapsone administration can produce hypersensitivity reactions in the form of severe cutaneous adverse reactions (SCARs). These have been strongly associated with the presence of the HLA-B*13:01 haplotype in patients treated with dapsone [60].

#### 4.2.1. Glucose-6-phosphate dehydrogenase (G6PD)

The *G6PD* gene encodes the cytosolic enzyme G6PD, responsible for metabolizing glucose-6-phosphate, transforming it into 6-phosphogluconolactone and producing NADPH; it is the first phase in the pentose phosphate pathway. This enzyme is expressed mainly in erythrocytes and, therefore, this reaction is one of the main sources of NADPH in erythrocytes [61].

*G6PD* is a polymorphic gene; more than 400 polymorphisms of a single nucleotide of *G6PD* have been described, and 186 of them are associated with the loss of G6PD enzyme activity and stability. The polymorphisms cause G6PD deficiency, manifested clinically as neonatal jaundice, acute hemolytic anemia, and chronic hemolytic anemia; however, most of the people who carry this genetic defect are asymptomatic. The *G6PD* gene is located on chromosome X and, therefore, the genetic alterations will be transmitted by sex-linked recessive inheritance; that is, men are classified as G6PD normal or deficient whereas women are classified into three G6PD phenotypes: normal, intermediate, and deficient. It is estimated that more than 400 million people have G6PD deficiency worldwide, making it the most common human enzymopathy. For this reason, enzyme activity tests are performed to classify G6PD deficiency phenotypically. However, patients with normal enzyme activity develop hematologic toxicity in the same way. This interindividual variability in dapsone toxicity may be due to genetic causes. The most extensively studied polymorphisms are those that reduce G6PD activity, which are most common in Africans, mainly in malaria-endemic areas. For this reason, most of the studies have been conducted in patients of this type [62]. In particular, a study carried out in a sub-Saharan African population with 117 cases and 234 controls considered the triallelic *G6PD* gene [59]. The *G6PD* type B allele is the most common variant worldwide, with normal enzyme activity. The *G6PD* type A+ allele, rs1050829, A376G (Asn → Asp), maintains 85% of enzyme activity and is, therefore, moderately deficient, and the *G6PD* type A− allele, rs1050828 A376G and G202A (Val → Met) reduces enzyme activity to 12% and is considered severely deficient. In tropical Africa, the *G6PD* type A− variant accounts for 90% of all G6PD deficiencies [59].

On the other hand, dapsone can be administered by a topical cutaneous application in low concentrations to treat acne vulgaris. A study with 56 Caucasian patients from the United States diagnosed with acne vulgaris and treated with topical dapsone evaluated the risk of hemolysis and/or hemolytic anemia in patients with G6PD enzyme deficiency [63]. Although the results of this study showed that treatment with topical dapsone in low concentrations did not involve a clinically significant risk of hemolysis or anemia in these patients, the FDA recommends avoiding the administration of this drug in patients with G6PD deficiencies [64].

#### 4.2.2. Major Histocompatibility Complex, Class I, B (HLA-B)

The *HLA-B* gene is located on chromosome 6p21.33 and forms part of the major histocompatibility complex (MHC). The HLA-B protein, one of the human leukocyte antigens, interacts particularly with NK and T cells and their receptors [65].

Dapsone binds to the Ile95 residue of the HLA-B protein, altering the structure of the antigen recognition site. This causes the recognition of its own ligands as external agents and gives rise to SCARs. This hypersensitivity reaction characteristically presents with fever, skin rash, and internal organ involvement in the 4–6 weeks after initiating dapsone treatment [66].

There are no studies evaluating the impact of the HLA-B*13:01 haplotype on DH. However, a study conducted in 872 Asian patients with leprosy treated with dapsone found that the presence of the HLA-B*13:01 allele was useful as a risk predictor for dapsone hypersensitivity syndrome (with a sensitivity of 85.5% and specificity of 85.7%) [67]. According to these results, patients carrying two copies of the allele have a greater risk of suffering hypersensitivity reactions compared to patients carrying a single allele. However, those with no copy of the allele have a very low or almost non-existent risk of SCARs. This study, therefore, suggests excluding dapsone as part of treatment in patients who carry one or more copies of the HLA-B*13:01 allele to reduce the risk of developing hypersensitivity reactions [67].

### 4.3. Biologic Drug Treatment

Biologic treatments represent a heavy economic burden for health care systems. Being able to determine the probability of a response and the potentially associated benefits before starting a biologic treatment brings us closer to a personalized health care and precision medicine model, obtaining a positive effect on the patient’s quality of life, on the one hand, by avoiding treatments with suboptimal effects that do not control the symptoms and allow the disease to progress, and on the other, a beneficial effect on the economy of the health care system. Failure of biologic treatment involves high costs, since starting these treatments requires an induction phase in which high doses are administered [68].

#### 4.3.1. Moderate-to-Severe Psoriasis

The treatment of moderate-to-severe psoriasis is based fundamentally on biologic therapies. Anti-TNFs, etanercept and adalimumab, and the IL12/IL23 inhibitor ustekinumab are regarded as first-line biologics [33]. Second-line treatments include the IL17 inhibitors, secukinumab, ixekizumab, and brodalumab [69,70]. In addition, guselkumab, tildrakizumab, and the recently approved risankizumab inhibit IL23, preventing interaction with the IL23 receptor complex [71,72]. With respect to the efficacy and toxicity of biologic therapies, head-to-head clinical trials have demonstrated that IL17 and IL23 inhibitor drugs are more effective than IL12/IL23 inhibitors and anti-TNFs [73]. However, the IL17 inhibitors, BDL, IXE, and SCK, are those with the highest probability of maintaining long-term efficacy (40–64 weeks) (97%, 83%, and 77%, respectively) [74]. As for safety, all of them have proved to be very safe; patients experience no increase in severe infection rates or internal malignancies [75]. It should be emphasized that UTK and SCK have the lowest rates of adverse effects (compared to other anti-TNF biologic therapies), even in patients with comorbidities in the case of SCK [34,76].

Despite the proven efficacy and safety, not all patients have good results; in some, the expected response is not obtained in the induction phase (16–24 weeks with the treatment) or they experience a loss of response in the maintenance phase (from 24 weeks to years with the treatment). Furthermore, certain patients suffer various degrees of toxicity [77]. This variability in the short- and long-term response, as well as the toxicity, may be due to genetic factors. Alterations in the genes involved in the pathologic environment of the disease, metabolism, or mechanism of action may influence the effectiveness of these drugs [78].

The allelic variants of the HLA genes have been extensively studied in PGx, but the results are contradictory [79,80,81,82,83]. The HLA proteins are part of the MHC and contribute by identifying exogenous proteins that may trigger an immune response. The HLA system is located at the PSORS1 locus on chromosome 6 and encodes a large number of HLA proteins with various functions [84]. The *HLA-A/TRAF3IP2* (rs9260313/rs13190932) and HLA-Cw*06/LCE3C_LCE3B del/ins haplotypes, together with the *HLA-B/MICA* (rs13437088) and *HLA-C* (rs12191877, rs1048554, rs610604) polymorphisms, have shown an association with a response to anti-TNF drugs [7,85,86,87]. However, the presence of the HLA-C*06:02 allele has shown an association with a response to UTK, but not to anti-TNF drugs [79,80,81,83,88,89]. Participating in the development and maintenance of psoriasis are cytokines, receptors, and other associated proteins which are the targets of biologic drugs. Therefore, alterations in the genes that encode those proteins are directly related to the response to these drugs. More than 200 variants of the tumor necrosis factor (*TNF*) gene have been identified, four of which are the most extensively studied in psoriasis because of their relationship to its physiopathology. In addition, *TNF* is the target of three biologics (ADA, INF, and CTL) indicated for the treatment of moderate-to-severe psoriasis. Consequently, genetic alterations in *TNF* may affect the response to these drugs (64). The *TNF-308* (rs1800629), *TNF-238* (rs361525), and *TNF-857* (rs1799724) variants have been associated with the risk of suffering from psoriasis and with the response to anti-TNFs in psoriasis and other autoimmune diseases, such as ankylosing spondylitis and Crohn’s disease [79,90,91]. Similarly, the *TNF-1031* (rs1799964) polymorphism, located in the promoter region of the *TNF* gene, has been associated with anti-TNF response [88,91]. The genetic alterations of the TNF alpha induced protein 3 (*TNFAIP3*) gene have also been extensively studied in various inflammatory pathologies. However, in patients with psoriasis treated with anti-TNFs and UTK, the effect of only two polymorphisms, *TNFAIP3* rs610604 and rs6920220, has been studied, with contradictory results [81,92,93,94].

On the other hand, there are two known genetic alterations in the interleukin 1 beta (*IL1B*) gene, rs1143623 and rs1143627, associated with the efficacy of the anti-TNF treatment and UTK [95]. Furthermore, the rs1800795 polymorphism in the interleukin 6 (*IL6*) gene, which encodes the IL6 cytokine, has been associated with a response to the anti-TNF treatment [96]. Additionally, two polymorphisms in the interleukin 12B (*IL12B*) gene, rs2546890 and rs3213094, have shown an influence on the response to biologic drugs, especially to UTK, an IL12/23 inhibitor [85,86,93]. Similarly, the impact of IL17 on certain autoimmune and inflammatory diseases, such as psoriasis, has given rise to the development of three drugs aimed at blocking these cytokines (SCK, IXE, and BDL) [97]. Recent studies have shown an association between polymorphisms of the IL17 genes (*IL17F* rs763780 and *IL17RA* rs4819554) and the response to the anti-TNF treatment and UTK [98,99,100]. Finally, in the interleukin 23 receptor (*IL23R*) gene, the *IL23R* rs11209026 polymorphism has shown an influence on the response to anti-TNF drugs in naive patients and on the risk of developing toxicity and/or paradoxical psoriasis through the anti-TNF treatment [88,101].

The Toll-like receptor (TLR) family are transmembrane proteins with a fundamental role in the immune response. The influence of polymorphisms of *TLR2* (rs4696480 and rs11938228), *TLR5* (rs5744174), and *TLR9* (rs352139) on treatment response has been demonstrated in Caucasian patients (from Denmark) diagnosed with moderate-to-severe psoriasis and treated with anti-TNF drugs (*n* = 376) and with UTK (*n* = 230) [95].

Finally, only one study has been conducted evaluating the association between genes involved in the pathologic environment of the disease, metabolism or mechanism of action and toxicity of biologic therapies. Specifically, polymorphisms of the *CTLA4* (rs3087243), *FBXL19* (rs10782001), *SLC12A8* (rs651630), and *TAP1* (rs1800453) genes have shown an association with susceptibility to developing toxicity and/or paradoxical psoriasis due to anti-TNF drugs [101].

#### 4.3.2. Hidradenitis Suppurativa

The treatment of this disease is a crucial challenge to avoid severe complications [102]. Recently, the use of ADA and INF has been approved in patients who do not respond to systemic drugs or in whom these are contraindicated, due to the satisfactory results shown in phase III clinical trials (PIONER I and II) [44].

There are many PGx studies evaluating the efficacy of biologic therapy in various pathologies [7]. However, in the case of HS, only two studies have been conducted in patients with moderate-to-severe HS [41,103]. The Savva et al. team evaluated the impact of various polymorphisms in the *TNF* and *TLR4* genes on the treatment response with anti-TNF drugs in 190 Caucasian patients (from Greece) diagnosed with HS, 32 of whom were treated with ADA [103]. It was observed that the polymorphism located in the promoter region of the *TNF* gene, *TNFα-238* rs361525, was associated with a susceptibility to and severity of the disease. However, the association between these polymorphisms and a response to the anti-TNF treatment was not statistically significant (*p* > 0.05) [100]. Subsequently, a genome-wide association study (GWAS) was conducted in the United States in patients diagnosed with HS with the aim of identifying the genetic variants associated with a response to ADA (*n* = 307) [41]. It was observed that the rs59532114 polymorphism of the *BCL2* gene was associated with a worse response to ADA, due to the fact that the *BCL2* gene encodes a regulator molecule of TNF inhibition in hair follicle tissues [41].

In addition, the association of HLA typing with a lack of response to ADA has been studied in Caucasian patients (from the United States and Europe), 269 diagnosed with HS and 365 with rheumatoid arthritis, treated with ADA. Only two HLA-DRB1 variants were found associated with a higher risk of developing anti-drug antibodies, which can lead to therapeutic failure [104].

## 5. Clinical Application of Each Drug

### 5.1. 5-Fluorouracil

5-FU is a drug administered intravenously, orally or topically, depending on the pathology. The enzyme dihydropyrimidine dehydrogenase (DPD) plays an important part in the metabolism of fluorouracil. Deficiency or reduced activity of this enzyme may lead to an accumulation of the drug [105].

There are clinical guidelines that recommended optimal doses, based on the genetic profile and the calculated *DPYD* activity score (*DYPD*-AS) [50]. The resulting value makes it possible to translate from genotype to phenotype, thereby establishing three metabolizing capacity profiles for the enzyme: normal, intermediate, and poor. It is worth emphasizing that the recommendations on which these guidelines are based focus on the variants, c.1905 + 1G > A (rs3918290), c.1679 T > G (rs55886062), c.2846A > T (rs67376798), and c.1129–5923C > G (rs75017182), with the greatest and clearest impact on DPD activity, its relationship to 5-FU clearance, and the toxicity produced after exposure to the medication [50]. The optimal dose recommendations established by the CPIC (Clinical Pharmacogenetics Implementation Consortium) indicate that patients with a normal metabolizer genotype (*DPYD*-AS: 2) can be treated with the conventional doses of the drug. For intermediate metabolizers (*DPYD*-AS: 1 or 1.5) the advice is to reduce the dose by 50%, while for poor metabolizers (*DPYD*-AS: 0 or 0.5), who are the most susceptible because of their high risk of severe toxicity, the main recommendation is to avoid the use of 5-FU. If this is not possible, the most suitable course is to administer a greatly reduced dose (a reduction of 75% in the standard reduced dose) combined with early therapeutic monitoring of the drug. It should be stressed that no reports are currently available offering evidence of the effectiveness of administering low doses of 5-FU to patients with this phenotype. A complete deficiency of the DPD function is rare, estimated at 0.01% to 0.5% in Caucasian individuals; partial deficiency has been estimated at between 3% and 8% of the Caucasian population [50].

In dermatology, 5-FU is used topically at low concentrations (0.5%) in patients diagnosed with AK and BCC; its systemic absorption is very low (6%) and it is, therefore, associated with a very low risk of toxicity [106]. Consequently, the health care authorities do not consider that this risk is associated with this route of administration and leave this decision to the discretion of the health professional [9]. However, several clinical cases of patients who have developed severe toxicity due to the administration of 5-FU at 5% or 0.5% have been described [106,107]. A 76-year-old patient diagnosed with BCC on the scalp and treated with 5-FU at 5% every 12 h developed a toxic syndrome characterized by diarrhea, abdominal pain, stomatitis, vomiting, and a skin rash, along with neutropenia and severe thrombocytopenia, triggering hospitalization in the first week of treatment. The medication was suspended and, finally, it was confirmed that this patient had a complete DPD enzyme deficiency [108]. On the other hand, a patient aged 69, diagnosed with AK of the lower lip, treated 1–2 times per week with topical 5-FU at 5% at the site of the lesion, developed severe neutropenia on the 11th day of treatment after 14 applications, and also began to suffer a loss of vision in the left eye days afterwards. He only improved after receiving treatment with subcutaneous filigastim, and the doctors, therefore, associated these episodes with 5-FU treatment. However, no determinations of the DPD enzyme were determined [106]. Similarly, a 64-year-old patient diagnosed with AK in the lower extremities, treated with 5-FU 5% in the form of an occlusive dressing, presented with severe adverse reactions (fever, shivering, erythematous eruption, and hepatitis) after a week of treatment. The occlusive dressing was, consequently, suspended, but application was maintained. In this case, no enzymatic determination of DPD was decided; however, the authors state that the symptoms that the patient developed were due to a toxic reaction to 5-FU at 5% [107]. Most of the published cases have been with a 5-FU at 5%; however, a case with 5-FU at 0.5% has also been published. The patient, aged 64, diagnosed with AK, developed potentially life-threatening toxicity after a week of treatment, presenting with extreme lethargy, fatigue, fever, and mouth erosions, as well as painful mucositis and other systemic side effects. Despite the relationship to 5-FU toxicity, it was not possible to associate it with DPD deficiency, since the patient refused to undergo enzyme deficiency genetic profiling [109].

In conclusion, the determination of genetic polymorphisms in the *DYPD* gene is crucial to selecting patients at risk of toxicity from topical 5-FU.

### 5.2. Dapsone

Dapsone is administered orally and topically. There are clinical studies demonstrating that treatment with oral dapsone has produced dosage-related hemolysis and hemolytic anemia and that people with a deficiency of the G6PD enzyme are at a greater risk.

Although there are currently no PGx recommendations included in clinical guidelines for this medication, competent health care authorities in various countries, such as the FDA, have introduced warnings in the summary of product characteristics for topical dapsone [64]. They state that precautions must be taken during the administration of the drug, specifying that susceptibility is greater depending on the G6PD genotype/phenotype, specifically if dapsone is combined with other medications or when it is administered at high doses, and they, therefore, stress that red blood cell or hemoglobin levels must be strictly monitored in these patients during treatment [110,111].

Although the allele frequency of these genetic alterations is low in Europe, the levels of this enzyme are determined per protocol in all patients before the administration of certain oxidizing agents, such as dapsone. However, genotyping of the G6PD enzyme has not yet been performed to determine which type of allele (B/A+/A−) is crucial to avoiding adverse reactions to oxidizing medications.

### 5.3. Biologic Drugs

There are currently no PGx recommendations applied in clinical practice for treatment with biologic therapies in patients diagnosed with psoriasis or HS. The association of the genetic polymorphisms described with the response or toxicity, therefore, needs to be validated in a larger number of studies, with larger patient cohorts and more uniform response criteria, to corroborate those associations [7].

The PharmGKB pharmacogenetic database compiles, selects, and disseminates PGx information from multiple sources, including the scientific literature, the dosage guidelines from clinical practice guides, and the PGx recommendations for certain medicines [112]. In this database, genetic polymorphisms are classified in different levels of evidence. The *TNF-308* rs1800629 polymorphism is the one with the strongest evidence; it has been extensively studied in patients treated with biologic drugs, specifically etanercept, and diagnosed with autoimmune conditions such as psoriasis, rheumatoid arthritis, and Crohn’s disease, showing moderate evidence (level 2B). In particular, Caucasian patients carrying the G allele obtained a better response to anti-TNF drugs compared to Caucasian patients carrying the A allele [113,114,115,116,117,118,119]. However, the other genetic polymorphisms studied have evidence level 3. In other words, associations between the drug and the genetic variant are considered on the basis of a single significant study (not yet replicated) or there are multiple studies but without clear evidence of an association.

On the other hand, the genetic polymorphisms related to the response to ADA in HS show very limited evidence. Only two PGx studies have been conducted in a Caucasian population (*n* = 339). This is due to the limited use of biologic therapies in HS; only recently has ADA been authorized for the treatment of moderate-to-severe HS [41].

In the near future, the study of biomarkers will help to predict and prognosticate the response to and/or toxicity of biologic therapies in moderate-to-severe psoriasis and hidradenitis suppurativa, making it possible to individualize the treatment according to the genetic profile of each patient.

## 6. Future Prospects

Recently, progress has been made in overcoming barriers to implementing PGx in regular clinical practice [11]. The immediate availability and decreasing cost of sequencing and genotyping technology have led to a considerable increase in the number of studies evaluating associations between human genetic variability and drug phenotypes, increasing the scientific evidence. The treatments of the pathologies described above have been the most extensively studied, and significant PGx findings have been obtained. However, not only has the impact of PGx been important in AK, BCC, DH, HS, and psoriasis, it may also play a crucial role in other dermatologic pathologies, such as keloid scars, erythema nodosum leprosum, bullous pemphigoid, dermatomyositis, and melanoma, among others [120,121,122]. Furthermore, a European project entitled Ubiquitous Pharmacogenomics (U-PGx) has recently been set up; it will enroll 8100 patients from seven European countries (Greece, Slovenia, Spain, the United Kingdom, the Netherlands, Austria, and Italy) to evaluate the utility of preventive PGx together with the cost-effectiveness and the incidence of interruption of the drug according to the guidelines of the Dutch Pharmacogenetics Working Group (DPWG) compared to the standard of care [123]. The challenge of using this genetic information to make decisions using clinical guidelines, such as those provided by the CPIC or DPWG, will be met as genotyping and genome sequencing become available in regular clinical practice.

On the other hand, the limited time available for decision-making by clinical staff to be able to deal with all the medical consultations that arise slows down the process of involving PGx in regular clinical practice. Support tools incorporated into electronic health records (EHRs) have been proposed as a possible way of helping with decision-making. However, because of the diversity of EHR systems, creating support tools for decision-making is still a big challenge [11]. Several health systems are currently working to optimize the implementation of pharmacogenomic data in clinical practice by using decision-making support tools integrated into EHRs. The European U-PGx project is also planning to implement a standardized PGx decision-making support tool in several languages. The objective is to use a digital content management system as a central knowledge base that can interact with the various EHR systems. This implementation of systems in the clinical setting highlights the computing challenges, but also provides a basis for improvement and standardization [123].

Finally, it is worth drawing attention to the Translational Pharmacogenetics Program (TPP) of the National Institutes of Health (NIH) Pharmacogenomics Research Network for having monitored the implementation of PGx in the clinical routine of eight United States health systems over a five-year period (2011–16), showing various solutions to overcoming many barriers. In conclusion, this program highlighted the fact that the CPIC recommendations provide uniformity and coherence in the various organizations [124].

## 7. Conclusions

Fluorouracil is an antitumor drug administered both parenterally and orally in oncology and topically in dermatology to treat AK and BCC. The activity of DPD, the fundamental enzyme in 5-FU metabolism, is subject to interindividual variability. Numerous genetic variants were identified in the *DPYD* gene, the most important being rs3918290, rs55886062, rs67376798, and rs75017182, since these give rise to a substantial reduction in enzyme activity and increase the risk of severe adverse reactions; they can even lead to death. As a result of this very clear association, we find certain PGx recommendations in clinical practice guides; however, these recommendations have not been extrapolated to the topical administration of 5-FU in dermatology owing to its limited absorption. Nevertheless, a number of clinical cases of toxicity associated with the topical administration of 5-FU have been published. The determination of genetic polymorphisms in the *DYPD* gene is, therefore, crucial to selecting patients with a risk of toxicity due to 5-FU.

On the other hand, G6PD deficiency is the most common human enzymopathy. Patients diagnosed with DH who are treated with oral dapsone and have deficient G6PD activity may develop severe hematologic adverse reactions. For this reason, enzyme activity levels are currently determined by classifying patients phenotypically before they are treated. However, some patients with normal enzyme activity develop hematologic toxicity. In Africa, the *G6PD* gene is considered triallelic (A+/A−/B) according to the genetic polymorphisms it presents; those most affected are A+ and A−. Health authorities, such as the FDA, have included PGx recommendations in the summary of product characteristics for dapsone, a preliminary step to including them in clinical practice guides. A PGx study has been conducted in patients diagnosed with acne vulgaris, treated with topical dapsone and with deficient G6PD, showing that it does not represent a clinically significant risk of hemolysis or anemia; however, the FDA also recommends avoiding the administration of this drug even topically in patients with G6PD deficiencies. Furthermore, an association was demonstrated between the HLA-B*13:01 allele and the development of hypersensitivity reactions (SCARs) in Asian patients with leprosy treated with oral dapsone.

Finally, it is worth highlighting the treatment with biologic therapies indicated for pathologies such as HS or moderate-to-severe psoriasis, for their notable results; however, certain patients do not obtain the expected effect in the short or long-term and suffer various degrees of toxicity. The influence of polymorphisms in genes involved in the pathologic environment of the disease, metabolism or mechanism of action on the effectiveness of these drugs has been investigated in many studies. However, the level of evidence in PharmGKB is low and more validation studies need to be conducted to transfer this information to clinical practice.

In conclusion, the application of PGx to dermatology has not been as rapid as in other medical areas, such as oncology. The treatments of the pathologies described above have been the most extensively studied and significant PGx findings have been obtained. However, not only has the impact of PGx been important in AK, BCC, DH, HS, and psoriasis, it may also play a crucial role in other dermatologic pathologies. It is also vital to transfer PGx recommendations in other systemic drugs to clinical practice to avoid the development of cutaneous adverse reactions.

## Figures and Tables

**Table 1:** Gene polymorphisms involved in response or toxicity to dermatological treatment by PharmGKB.

Gene	Polymorphism	Drug	Pathology	Population	PharmGKB Level of Evidence	Clinical Application	Alelle	Phenotype Category	References
DYPD	rs3918290	5-FU	KA, CBC *	Multiple groups (Caucasian, Black/African American, Asian...)	1A	Yes	CC < CT < TT	Toxicity	[32,77]
	rs55886062	5-FU	KA, CBC *		1A	Yes	AA < AC < CC	Toxicity	[32,77]
	rs67376798	5-FU	KA, CBC *		1A	Yes	TT < TA < AA	Toxicity	[32,77]
	rs75017182	5-FU	KA, CBC *		1A	Yes	GG < GC < CC	Toxicity	[32,77]
G6PD	G6PD A- rs1050828	Dapsone	Malaria	Africans	1B	No	A376G y G202A	Toxicity	[80,81]
	G6PD A+ rs1050829	Dapsone	Malaria	Africans	-	No	A376G	Toxicity	
HLA-B	HLA-B*13:01:01	Dapsone	Leprosy	Asian	2A	No	(+)	Toxicity	[44]
TNF	rs1800629	Anti-TNF	Aps, AR, CD, SA **, Psoriasis	Caucasians (European)	2B	No	AA < AG < GG	Efficacy	[79,90,91]
	rs1799724	Anti-TNF	AR, CD, SA***	Asian, Caucasians (Spain)	4	No	TT + CT < CC	Efficacy	[79,90,91]
TNFAIP3	rs610604	Anti-TNF	Aps^+^, Psoriasis	Caucasians (Italy, Netherlands, Spain)	3	No	GG < TT	Efficacy	[94]
IL1- β	rs1143623	Anti-TNF/UTK	Psoriasis	Caucasians (Denmark)	3	No	GG + CG < CC	Efficacy	[95]
IL1- β	rs1143627	Anti-TNF/UTK	Psoriasis		3	No	GG + AG < AA	Efficacy	[95]
Il-6	rs1800795	Anti-TNF	Psoriasis	Caucasians (Italy)	3	No	GG < CG < CC	Efficacy	[96]
IL12-β	rs2546890	Anti-TNF	Psoriasis	Caucasians (Spain)	3	No	GG + AG < AA	Efficacy	[86]
IL12-β	rs3213094	UTK	Psoriasis	Caucasians (Netherlands)	3	No	CT < CC	Efficacy	[93]
IL23R	rs11209026	Anti-TNF	Psoriasis	Caucasians (Spain)	3	No	GG < AG	Toxicity	[88,101]
TLR2	rs4696480	Anti-TNF	Psoriasis	Caucasians (Denmark)	3	No	TT + AT < AA	Efficacy	[95]
	rs11938228	Anti-TNF	Psoriasis, Inflammatory bowel diseases		3	No	AA + AC < CC	Efficacy	[95]
TLR5	rs5744174	UTK	Psoriasis		3	No	AA < A + GG	Efficacy	[95]
TLR9	rs352139	Anti-TNF	Psoriasis		3	No	CC < CT + TT	Efficacy	[95]
BCL2	rs59532114	ADA	Hidradenitis Suppurativa	Caucasians (American)	-	No	CC > CA + AA	Efficacy	[41]

* KA, CBC: actinic keratosis and basal-cell carcinoma; ** Aps, AR, CD, SA: Arthritis Psoriatic, Arthritis Rheumatoid, Crohn Disease, Spondylitis Ankylosing; *** AR, CD, SA: Arthritis Rheumatoid, Crohn Disease, Spondylitis Ankylosing; ^+^ Aps: Psoriatic Arthritis; 5-FU: 5-Fluorouracil; Anti-TNF: inhibitors TNF drugs; UTK: ustekinumab; ADA: adalimumab.

## Data Availability

Data sharing not applicable.
